# Arginase 2 Deletion Reduces Neuro-Glial Injury and Improves Retinal Function in a Model of Retinopathy of Prematurity

**DOI:** 10.1371/journal.pone.0022460

**Published:** 2011-07-21

**Authors:** Subhadra P. Narayanan, Jutamas Suwanpradid, Alan Saul, Zhimin Xu, Amber Still, Robert W. Caldwell, Ruth B. Caldwell

**Affiliations:** 1 Vascular Biology Center, Georgia Health Sciences University, Augusta, Georgia, United States of America; 2 Department of Cellular Biology and Anatomy, Georgia Health Sciences University, Augusta, Georgia, United States of America; 3 Department of Ophthalmology, Georgia Health Sciences University, Augusta, Georgia, United States of America; 4 Department of Pharmacology and Toxicology, Georgia Health Sciences University, Augusta, Georgia, United States of America; 5 Charlie Norwood VA Medical Center, Augusta, Georgia, United States of America; University of Bristol, United Kingdom

## Abstract

**Background:**

Retinopathy of prematurity (ROP) is a major cause of vision impairment in low birth weight infants. While previous work has focused on defining the mechanisms of vascular injury leading to retinal neovascularization, recent studies show that neurons are also affected. This study was undertaken to determine the role of the mitochondrial arginine/ornithine regulating enzyme arginase 2 (A2) in retinal neuro-glial cell injury in the mouse model of ROP.

**Methods and Findings:**

Studies were performed using wild type (WT) and A2 knockout (A2−/−) mice exposed to Oxygen Induced Retinopathy (OIR). Neuronal injury and apoptosis were assessed using immunohistochemistry, TUNEL (terminal deoxynucleotidyl transferase dUTP nick end) labeling and Western blotting. Electroretinography (ERG) was used to assess retinal function. Neuro-glial injury in WT ROP mice was evident by TUNEL labeling, retinal thinning, decreases in number of rod bipolar cells and glial cell activation as compared with room air controls. Significant reduction in numbers of TUNEL positive cells, inhibition of retinal thinning, preservation of the rod bipolar cells and prevention of glial activation were observed in the A2−/− retinas. Retinal function was markedly impaired in the WT OIR mice as shown by decreases in amplitude of the b-wave of the ERG. This defect was significantly reduced in A2−/− mice. Levels of the pro-apoptotic proteins p53, cleaved caspase 9, cytochrome C and the mitochondrial protein Bim were markedly increased in WT OIR retinas compared to controls, whereas the pro-survival mitrochondrial protein BCL-xl was reduced. These alterations were largely blocked in the A2−/− OIR retina.

**Conclusions:**

Our data implicate A2 in neurodegeneration during ROP. Deletion of A2 significantly improves neuronal survival and function, possibly through the regulation of mitochondrial membrane permeability mediated apoptosis during retinal ischemia. These molecular events are associated with decreased activation of glial cells, suggesting a rescue effect on macroglia as well.

## Introduction

Retinopathy of prematurity (ROP) is a major cause of childhood vision impairment in developed countries. Despite the improvements in neonatal care, the incidence of ROP does not decline, probably due to increased survival of premature infants. From the clinical perspective ROP is considered as a vascular disease and current treatments target abnormal retinal angiogenesis. However, despite effective treatment of the vascular injury, many children suffer vision impairment suggesting a disruption of neuronal development [1]. It has been shown that the age of onset of ROP coincides with the time of photoreceptor development [2]. Studies have reported that rod photoreceptors are affected [3], [4], [5] and visual acuity is reduced [6] in ROP patients. Deficits in rod and rod-bipolar cell sensitivity have been observed several years after ROP has resolved [5], [7]. Recent study has shown that the function of the post receptor neural retina is significantly impaired in a rat model of ROP [8], [9].

Oxygen induced retinopathy (OIR) in mice is a useful model for studies aimed at defining the molecular mechanisms of ROP. In addition to pathological neovascularization, retinal degeneration [10], [11], [12] and glial activation [12], [13] have been demonstrated. Apoptosis is reported to be the major mechanism of the retinal degeneration [11]. Upregulation of the constitutively active isoform of nitric oxide synthase (NOS), inducible NOS (iNOS) has been shown to be involved in triggering retinal apoptosis during OIR by a process involving formation of the highly toxic oxidant peroxynitrite [11]. Under normal physiological conditions NOS uses its substrate L-arginine to produce NO. However, if the supply of L-arginine is limited, NOS will use molecular oxygen to produce superoxide. Superoxide reacts rapidly with any available NO to form peroxynitrite. Thus, a deficiency in L-arginine supply can lead to increased peroxynitrite formation. L-arginine is also the substrate for the urea/ornithine-producing enzyme, arginase. Arginase has been shown to limit the normal function of NOS in aging and disease conditions, including hypertension, ischemia-reperfusion injury and diabetes [14]. Increases in arginase activity and arginase 2 mRNA have been reported in the OIR mouse model [15]. Given the involvement of increased arginase activity in altering NOS function in other disease conditions and the demonstrated involvement of iNOS in retinal apoptosis, arginase could play role in retinal injury associated with OIR.

Arginase exists in two forms, arginase 1 and 2, encoded by different genes. Arginase 1 (A1) is localized in the cytoplasm and expressed most abundantly in the liver, while arginase 2 (A2) is a mitochondrial enzyme, expressed primarily in extrahepatic tissues [16]. Studies conducted in our laboratory have shown that, in retina, arginase 1 is expressed in the retinal glia and microglia [17]. However, the localization of arginase 2 in the retina is not yet known. The present study is aimed at investigating the role of arginase 2 in neural cell injury during OIR and its impact on retinal function.

## Results

### Deletion of Arginase 2 reduces retinal degeneration during OIR

**Figure 1:**
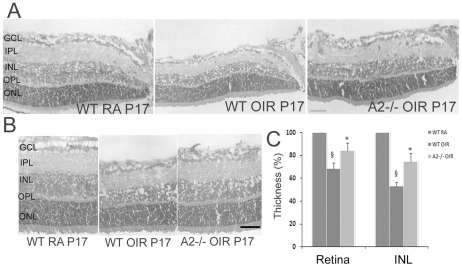
Retinal degeneration in WT and A2−/− mice during OIR. A) H & E staining of retinal sections from WT RA, WT OIR and A2−/− OIR at P17 showing the markedly reduced thickness of retina and INL in WT OIR compared to WT RA, and increased thickness of retina and INL in A2−/− compared to WT OIR at P17. Scale bar 50 µm. B) Representative images used for quantifying the thickness of retina and INL. Scale bar 100 µm. C) Quantification of retinal and INL thickness. Histogram showing the relative changes in thickness of retina and INL during OIR, quantified using NIH ImageJ. In comparison with WT RA (100%), thickness of both retina and INL is significantly reduced in WT OIR (P<0.01), while they are significantly increased in A2−/− OIR compared to WT OIR. (P<0.01), Data presented as mean±SD. N = 6. § WT RA vs WT OIR, * A2−/− OIR vs WT OIR. GCL, ganglion cell layer; IPL, inner plexiform layer; INL, inner nuclear layer; OPL, outer plexiform layer; ONL, outer nuclear layer.

In addition to neovascularization, retinal thinning is a major feature of OIR [11]. In the current study, thickness of the retina and inner nuclear layer (INL) at P17 (post natal day 17) was measured using H&E stained sections. As shown in Figure 1, thickness of retina and INL were significantly reduced in WT OIR compared to room air (RA) controls (p<0.01), while in A2−/− OIR, thickness of both retinal and INL was significantly preserved (p<0.01) in comparison with WT OIR. Retinal degeneration was also studied in double knockout mice lacking one copy of A1 as well as both copies of A2 (A1+/− A2−/−, deletion of both copies of A1 is lethal at P10–14). Both retinal and INL thickness were comparable to that in the A2−/− OIR mice (data not shown). These results demonstrate that A2 plays an important role in the neurodegeneration during OIR.

### Arginase 2 expression is altered during OIR

**Figure 2:**
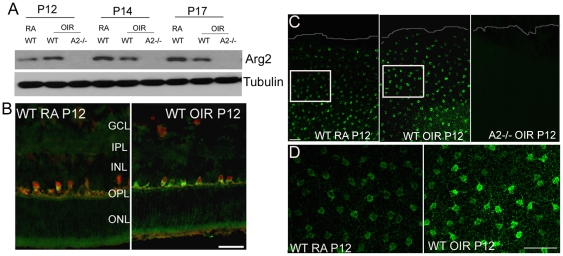
Expression of arginase 2 during OIR. A) Western blot analysis showing changes in the expression of A2 during OIR. Increased expression in WT OIR was observed at P12. B) Immunohistochemistry of retinal cryostat sections at P12 showing A2 (green) expression in horizontal cells. Calbindin (red) is used as a marker for horizontal cells. Scale bar 100 µm. C) Low magnification confocal images of retinal flat mounts (periphery is outlined) from WT RA, WT OIR and A2 −/− OIR at P12 immunostained for A2 showing enhanced levels in the horizontal cells in WT OIR. White boxes represent area for high magnification images in panel D. Scale bar 100 µm. D) High magnification images of WT RA and WT OIR retinal flatmounts at P12 immunostained for A2, demonstrating increased A2 expression in the horizontal cells of WT OIR compared with WT RA. Scale bar 50 µm. A minimum of three animals was used for each experiment and representative images are shown. GCL, ganglion cell layer; IPL, inner plexiform layer; INL, inner nuclear layer; OPL, outer plexiform layer; ONL, outer nuclear layer.

A2 is reported to be localized in mitochondria and is known to be expressed in a variety of tissues including retina [16], but so far its specific cellular localization in the retina is unknown. In brain tissue A2 is reported to be localized in neurons and glia [18]. In the present study, experiments were performed to examine the expression and localization of A2 in mouse retinal samples using Western blotting and immunohistochemistry methods. As evident from Figure 2, increased expression of A2 was observed at P12 in WT OIR samples compared to WT RA controls. However, relative levels of A2 protein were slightly reduced in the WT OIR retina at later stages (Fig. 2A). There was an age dependant increase in A2 levels in control samples which was not observed in the OIR retinas. Immunolocoalization analysis of A2 distribution at P12 in the WT retina showed high levels of expression in horizontal cells as compared to other retinal cell types (Fig. 2B). A population of A2 positive cells in the outer margin of the INL was also strongly immunoreactive for the horizontal cell marker calbindin (Fig. 2B). Immunostaining of retinal flatmouts using arginase 2 antibody clearly demonstrated increased expression of A2 in the horizontal cells of WT OIR retinas (Fig 2C and D). These results demonstrate that a primary site of A2 distribution is in horizontal cells and its expression is increased at the start of the ischemic phase of OIR. Analysis of arginase activity using an assay for urea formation showed no difference between the WT OIR and WT RA controls at P12, P14 or P17 (Table 1). This apparent lack of an effect of the oxygen treatment on arginase activity is probably due to the fact that the assay measures activity of both arginase isoforms. A further complicating factor is that arginase 2 immunoreactivity is localized mainly in horizontal cells, which are relatively few in number compared with other retinal cell types, whereas arginase 1 is distributed in the Muller glia, which are abundant in the retina.

**Table 1:** Arginase activity in retinal samples.

Age	WT RA	WT OIR
P12	209.3±7.6	210.4±36.5
P14	264.6±7.5	232.0±22.0
P17	265.0±31.2	264.4±5.6

Arginase activity was analyzed in WT RA and WT OIR retinal samples at P12, P14 and P17 and is shown as nmol/mg protein/min. A minimum of 3 animals were used in each group and data are presented as mean ± SEM.

### OIR-induced apoptosis is reduced in A2−/− retina

**Figure 3:**
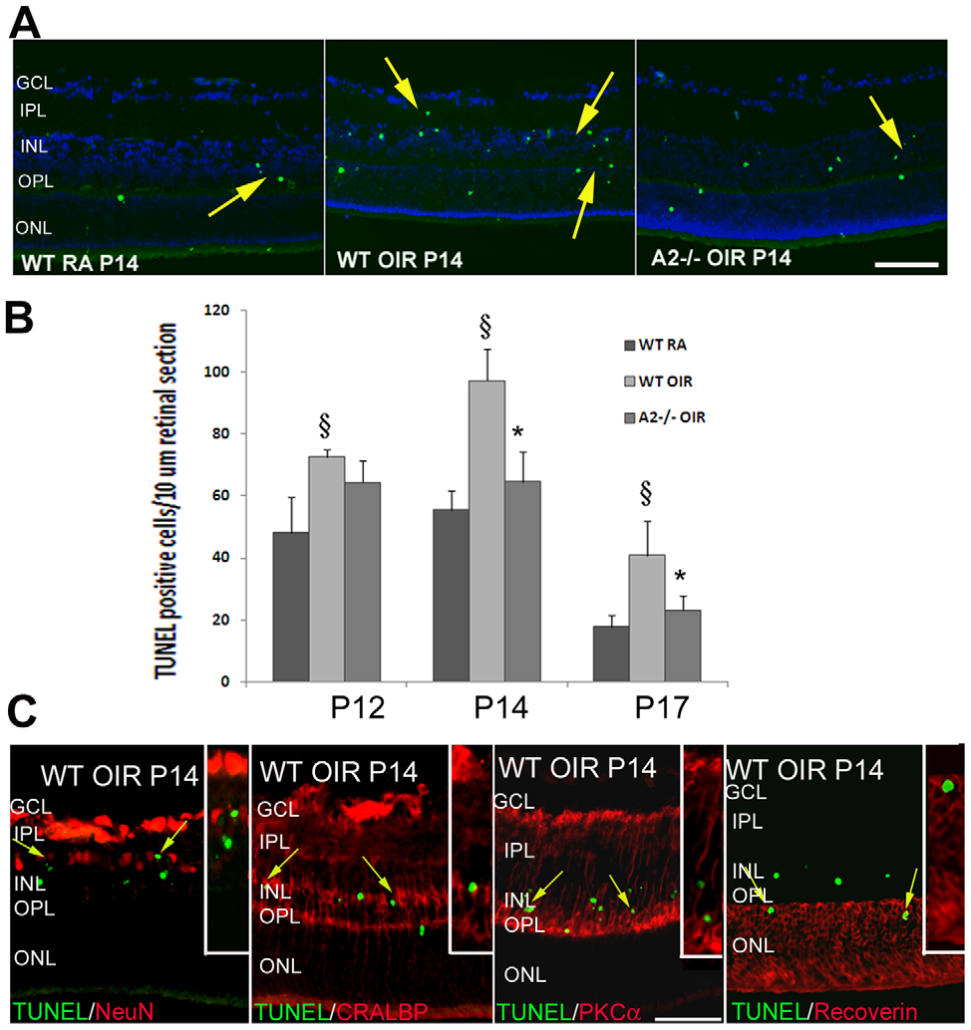
Decreased apoptosis in A2−/− OIR retina. **A**) TUNEL assay of retinal sections showing the presence of numerous apoptotic cells (colocalized with DAPI) in WT OIR compared to room air (RA) controls and A2−/− OIR at P14. B) Quantification of TUNEL positive cells showing significantly increased number in WT OIR at all stages. Numbers of apoptotic cells were significantly reduced in A2−/− OIR compared to WT OIR. Data presented as mean±SD. C) Characterization of TUNEL positive cells on WT OIR retinal cryostat sections at P14, by double immunolabelling using NeuN (ganglion and amacrine cells), CRALBP (Muller glia), PKCα (bipolar cells) and recoverin (photoreceptors) antibodies. Insets show magnified images of TUNEL colocalization with respective cellular markers. N varies from 4–6. §WT RA vs WT OIR (P<0.01), * A2−/− OIR vs WT OIR (P<0.05). Scale bar 100 µm. GCL, ganglion cell layer; IPL, inner plexiform layer; INL, inner nuclear layer; OPL, outer plexiform layer; ONL, outer nuclear layer.

Apoptosis is reported to be the major mechanism of retinal degeneration during OIR [11]. In the present study, retinal apoptosis was studied in WT RA, WT OIR and A2−/− OIR mice on P12, P14 and P17. At all stages, the number of TUNEL (terminal deoxynucleotidyl transferase dUTP nick end labeling) positive cells was reduced in A2−/− OIR compared to WT OIR retinas. Further characterization of TUNEL positive cells using double immunolabeling with neuronal and glial markers confirmed that cells undergoing apoptosis in the OIR retina included amacrine cells (NeuN positive), bipolar cells (PKCα positive), Muller glia (CRALBP positive) and photoreceptors (recoverin positive) Numerous TUNEL-positive apoptotic cells were observed in WT RA sections mainly at P12 and P14, which is due to developmental apoptosis. The peak of apoptosis during OIR was observed to be at P14 (Fig. 3A). The number of apoptotic cells was significantly reduced in A2−/−OIR compared to WT OIR at P14 and P17 (Fig. 3B). This further confirms that deletion of A2 reduces neurodegeneration in the OIR retina.

### OIR-induced glial activation is prevented in A2−/− retina

**Figure 4:**
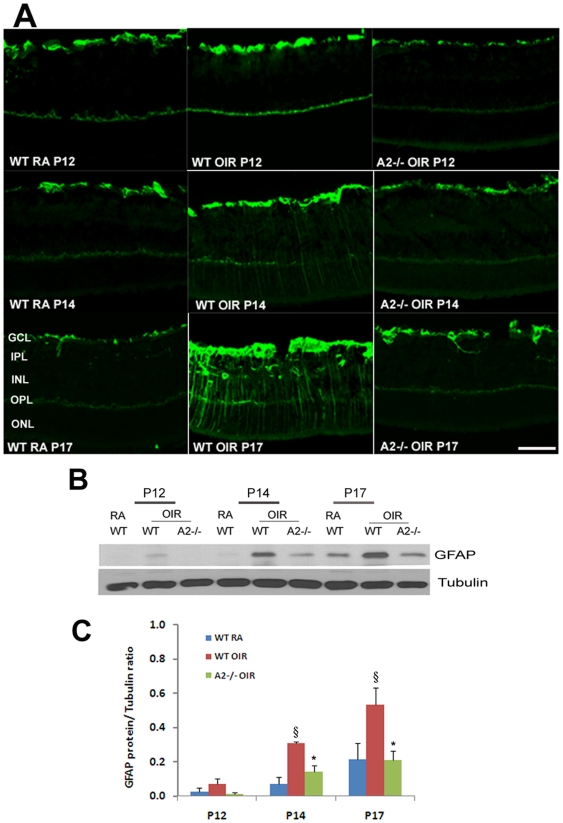
Decreased glial activation in A2−/− OIR retina. A) Immunohistochemistry of retinal cryostat sections from WT RA, WT OIR and A2−/− OIR using anti- GFAP antibody at different stages of hypoxic phase. B) Western blot analysis using retinal homogenates showing the changes in GFAP expression in WT OIR retina compared to RA and A2−/− OIR during different stages. C) Quantification of GFAP expression levels in WT RA, WT OIR and A2−/− OIR at P12, P14 and P17. Data presented as mean±SD. §WT RA vs WT OIR (P<0.01), *A2−/− OIR vs WT OIR (P<0.01), N varies from 4–6. Scale bar 100 µm. GCL, ganglion cell layer; IPL, inner plexiform layer; INL, inner nuclear layer; OPL, outer plexiform layer; ONL, outer nuclear layer.

Activation of glial cells is a common feature in the OIR retina [12], [13] and is characterized by increases in the expression of glial fibrillary acidic protein (GFAP). In normal conditions, GFAP is expressed by retinal astrocytes whereas following retinal injury, GFAP is expressed by Muller cells as well. Muller cells are radial glia whose processes span the retina extending from the nerve fiber layer to the photoreceptor layer. Figure 4A shows that there was a slight increase in the levels of GFAP in the WT OIR retina as early as P12 compared to the WT RA. The enhanced expression of GFAP in Muller cells was more evident at P14 in WT OIR and was further increased at P17 as indicated the strong immunoreactivity extending radially from the inner retina through the outer nuclear layer. However this enhanced level of GFAP expression was not observed in A2−/− OIR retina at any stage of OIR studied (Fig. 4A). Quantification of Western blots using Image J showed that the increase in the expression of GFAP level was significant in WT OIR at P14 and P17 (p<0.01) compared to WT RA as well as A2−/− OIR. Similarly, differences in the expression level of GFAP between WT RA and A2−/− OIR were not significant, confirming that injury to glial cells was minimal in A2−/− OIR retina.

### OIR-induced loss of rod bipolar cells is reduced in A2−/− retina

**Figure 5:**
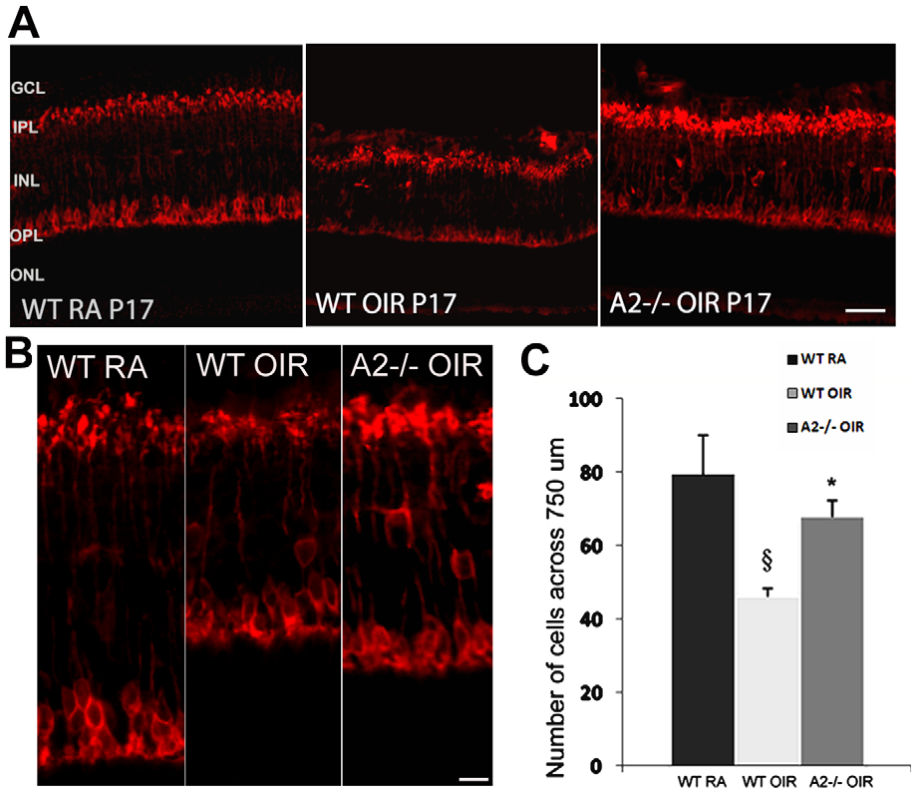
Preservation of bipolar cells in A2−/− OIR retina. A) Immunostaining of WT RA, WT OIR and A2−/−OIR retinal sections at P17 using rod bipolar cell marker PKCα. Scale bar 100 µm B) High magnification images of rod bipolar cells demonstrating the neurodegeneration of rod bipolar cells in WT OIR retina. Scale bar 25 µm. C) Quantification of PKCα positive cells showing significantly increased number in A2−/−OIR retina compared to WT OIR. Data presented as mean±SD. §WT RA vs WT OIR (P<0.01), * A2−/− OIR vs WT OIR (P<0.05), N varies from 3–4. GCL, ganglion cell layer; IPL, inner plexiform layer; INL, inner nuclear layer; OPL, outer plexiform layer; ONL, outer nuclear layer.

Bipolar cells are interneurons that transmit visual signals from the photoreceptors to the ganglion cells [19]. Function of rod bipolar cells is reported to be impaired in ROP patients [5], [7]. Decreases in numbers of bipolar cells have been reported in the rat model of OIR [20]. In the present work, we investigated the effect of A2 deletion on bipolar cell injury during OIR. Immunohistochemistry analysis of retinal sections using an antibody against the rod bipolar cell marker PKCα confirmed bipolar cell injury in WT OIR retina (Fig. 5A) as indicated by shorter and /or distorted axons and smaller cell bodies (Fig. 5B). Quantification of the number of PKCα positive cells showed that there is a significant reduction in rod bipolar cell number in WT OIR compared to WT RA at P17 (Fig. 5C). The number of rod bipolar cells in A2−/− OIR retina was not significantly different from that in the WT RA retina. These results demonstrate that rod bipolar cells are preserved in the A2−/− OIR retina.

### OIR-induced impairment of inner retinal function is reduced in A2−/− mice

**Figure 6:**
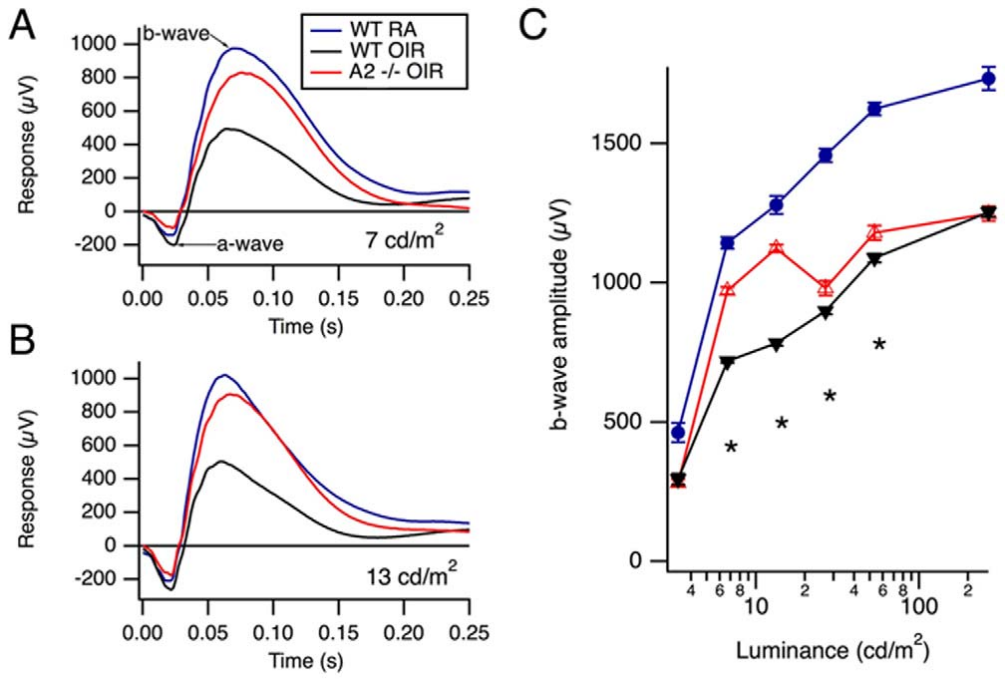
Retinal responses. A, B) ERGs recorded from 4 weeks old WT RA, WT OIR, and A2−/− OIR mice at intensities of 7 cd/m^2^ (candela per square meter) and 13 cd/m^2^ respectively. The data show averages of responses over both eyes, in 9, 6, and 16 WT RA, WT OIR, and A2−/− OIR mice, respectively. C) Mean b-wave amplitudes for WT RA, WT OIR, and A2−/− OIR mice are plotted against intensity. Responses are represented as mean ± SEM of the b-wave amplitudes, measured from the trough of the a-wave to the peak of the b-wave. Asterisks indicate significant differences between the two WT and A2−/− OIR groups (P<0.05, t-test). WT RA responses were significantly larger compared to both OIR groups at all intensities studied. Sample size varies from 6–16. * A2−/− OIR vs WT OIR (P<0.05).

The effect of OIR on retinal function in WT and A2−/− mice was analyzed using ERG. There have been reports of impaired retinal function in ROP patients [4], [5], [21] as well as in rat models of OIR [2], [8], [22]. These studies have shown decreases in the amplitude and sensitivity of the a-wave, suggesting photoreceptor dysfunction, as well as reductions in b-wave amplitudes, indicating that retinal interneurons are affected as well. Bipolar cell function (indicated by the b-wave amplitude) in WT-OIR was shown to be markedly impaired compared to WT RA (Fig. 6 A, B and C), while this defect was partially prevented in A2−/− OIR mice. The b-wave amplitudes in A2−/− OIR mice were significantly higher than in WT OIR at lower light intensities, as shown in Figure 6, demonstrating improved retinal function in A2−/− OIR mice. However at higher intensities the responses were similar in the two OIR groups. These observations are consistent with our immunolocalization data showing significant preservation of the rod bipolar cells in the A2−/− OIR mice at P17, compared to WT OIR mice. Additionally, these data support our observation that the number of apoptotic cells in the INL of the A2−/− OIR is significantly reduced as compared with the WT OIR retinas.

### Neuro-glial protection in A2−/− retina involves decreases in p53 and cleaved caspase-9

**Figure 7:**
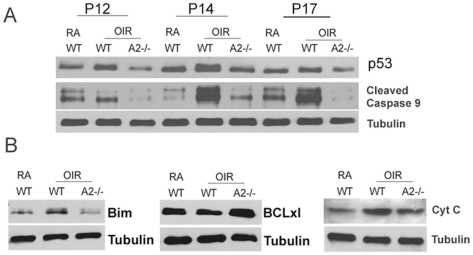
Signaling mechanism involved in A2-deficiency mediated neuro-glial protection in OIR retina. A) Western blot analysis showing the levels of p53, and cleaved caspase 9 in WT RA, WT OIR and A2−/− OIR at stages P12, P14 and P17 of the hypoxic OIR phase. B) Western blots showing changes in the expression of apoptotic markers Bim, BCLxl and cytochrome C in WT RA, WT OIR and A2−/− OIR at P14. N varies from 3–5, and representative images are shown.

Studies on potential mechanisms underlying the neuro-glial protection observed in the A2−/− OIR retina demonstrated the involvement of p53 mediated signaling mechanisms. The tumor suppressor and transcription factor p53 (protein 53) is a modulator of cellular stress responses and activation of p53 can mediate apoptosis in neurons [23], [24], [25]. The level of p53 was relatively elevated in WT OIR retinal homogenates at P12, P14 and P17 compared to WT RA with maximum increase at P14 (Fig. 7A). Additionally cleaved caspase 9 was shown to be significantly increased in WT OIR retina at P14. By contrast with the WT OIR retina, the levels of p53 and cleaved caspase 9 in the A2−/− OIR retina were always similar to RA controls. At P14, an increase in levels of the pro-apoptic marker Bim along with a decrease in survival marker BCLxl was also observed in WT OIR samples compared to WT RA controls, consistent with alterations in mitochondrial membrane permeability leading to cytochrome C release. However these changes were markedly abrogated in A2−/− OIR retina, demonstrating inhibition of apoptosis in these samples (Fig. 7B). These studies suggest that the OIR-induced neuronal injury and the resulting glial activation are regulated through an apoptotic pathway involving activity of A2, p53 and cleaved caspase 9 via alterations in mitochondrial membrane permeability.

## Discussion

These results identify a novel role of arginase 2 in retinal neuronal dysfunction associated with ROP. Abnormal vasculature characterized by vaso-obliteration and pathological angiogenesis is the most recognized characteristic of ROP. However the vascular pathology represents only one facet of the disorder. ROP results in retinal neuronal dysfunction as well [4], [5], [21], [26], [27]. However the cellular mechanisms of the neuronal injury are still unknown. In the present study we have investigated the impact of A2 deletion on neurodegeneration, glial activation and retinal function using a mouse model of OIR.

Retinal degeneration during OIR has been reported previously [11], [12]. Here we demonstrate that deletion of the A2 gene reduces the neuronal cell loss. Partial deletion of A1 along with A2 did not confer any additional protection, suggesting that A2 is the more important isoform of arginase involved in OIR-induced neurodegeneration. Sennlaub et al. [11] have previously shown that deletion of iNOS significantly reduces retinal thinning during OIR via reducing neurotoxicity associated with increases in protein nitration. Further study is required to determine the specific relationship between activity of arginase 2 and iNOS and peroxynitrite formation in relation to neuro-glial cell injury.

So far no literature is available on A2 localization in retina. Studies from our laboratory have shown A1 localization in glial cells [17]. In the current study we have demonstrated that A2 is most prominently expressed in horizontal cells and is present at lower levels in other cells of the inner nuclear layer. Our results are consistent with studies in brain, where A2 is shown to be present in neurons [18]. Our Western blot analysis showed that A2 protein levels were markedly increased immediately after the end of the hyperoxia exposure on P12 and decreased thereafter. Despite these alterations in A2 protein levels, total arginase activity remained constant. However, the arginase assay measures activity of both arginase isoforms. Given that the A2 expressing horizontal cells are relatively few in number compared with the A1 expressing retinal glial cells, it is likely that activity of arginase 1 masks the changes in arginase 2 activity.

Even though the horizontal cells are few in number, they are ideally situated and equipped to influence neuronal function and survival. As shown in Figure 2, their cell bodies are localized to a single layer, but are distributed across the entire retina. Their processes synapse with photoreceptor and bipolar cells and are also interconnected with other horizontal cells by gap junctions to form a network across the retina [28]. Moreover, the horizontal cells express neuronal NOS [29] and are involved in glutamate signaling [30]. Glutamate is an excitotoxic neurotransmitter and a product of the arginase / ornithine pathway [31]. Based on our finding that arginase 2 is expressed mainly in horizontal cells and that its deletion reduces loss of retinal neurons expressing markers for photoreceptor, bipolar and amacrine cells, we speculate that neuronal cell injury during oxygen-induced retinopathy involves arginase 2-induced increases in glutamate formation and dysregulation of neuronal NOS function. Further studies are needed to investigate these possibilities.

Based on our observation that apoptosis was maximal at P14, we hypothesize that oxygen-induced obliteration of the immature retinal vessels leads to relative retinal hypoxia/ischemia which increases expression/activity of A2 leading to death of retinal neurons. Similar to Sennlaub et al [11], we observed that the apoptosis peaked at P14 and the majority of the apoptotic cells were in the INL. The decrease in A2 at P14 and later is likely due to degeneration of A2 expressing cells. Lachapelle et al [27] have reported significant loss of horizontal cells in neonatal rats exposed to hyperoxia. It is also possible that the activity per amount of enzyme is higher irrespective of expression level and hence arginase activity could be present at the later stages of OIR even though total protein levels are reduced. This could lead to alterations in arginine metabolism resulting in a decrease in nitric oxide formation and/or increases in polyamines and/or glutamate. Polyamines have been implicated in exitotoxic neuronal death in retina [32] and the neurotoxic actions of glutamate are well established [33].

In addition to neovascularization and retinal thinning, activation of glial cells is another hallmark of ROP [12], [13]. This has been confirmed in our study. Absence of Muller glial activation in A2−/− OIR suggests an adverse effect of A2 on glia as well. Our data also demonstrate that glial activation follows neuronal apoptosis. While the peak of apoptosis was at P14, glial injury was greatest at P17. It is possible that the soluble factors released by cells undergoing death and/or cellular debris resulting from increased apoptosis are causing glial cell injury. According to Fletcher et al. [1], neurochemical changes observed during OIR could be attributed to changes in Muller cells. Our data further demonstrate that significantly reduced apoptosis in A2−/− OIR retinas is associated with decreases in glial cell activation. Downie et al. [12] have shown that up regulation of GFAP is restricted mainly to avascular and mid peripheral retina suggesting an involvement of relative retinal hypoxia secondary to the vascular injury. However, more detailed studies are required to examine whether neurodegeneration and/or vascular pathology underlie the macroglial activation observed in OIR.

The neuroprotective effect of A2 deletion was further confirmed by the ERG analysis showing improved retinal function in A2−/− OIR mice. Defects in retinal function as shown by ERG have been reported for rat OIR models [8], [9], [22]. However, our data are the first we are aware of showing functional losses in the mouse OIR model. Our finding of decreases in bipolar cell number during OIR is consistent with our observation of decreases in the b-wave amplitude of the ERG at low light intensity. Clinical studies have shown loss of neuronal function in ROP patients [3], [4], [6]. Deficits in rod and rod-bipolar cell sensitivity are still evident several years after ROP has resolved [5], [7]. The current treatment for ROP is laser photocoagulation or cryotherapy to destroy the ischemic tissue and reduce metabolic demand which promotes regression of the pathological blood vessels. Yet, many patients suffer from vision problems later suggesting loss of neuronal function. Since the development of laser surgery there has been little progress in the treatment of ROP. Based on increasing evidence that neurons as well as vascular endothelial cells are affected in ischemic retinopathies, it is suggested that retinal neurons are important targets in developing novel therapies for ischemic retinopathies.

It has been shown that NOS signaling is involved in retinal apoptosis during retinal ischemia related disorders [11], [34]. Several laboratories have reported that NO neurotoxicity is responsible at least in part for neural degeneration following retinal ischemia [34], [35]. Our current data show that deletion of the arginase 2 gene significantly reduces neurodegeneration associated with OIR. This beneficial effect could be through the regulation of NO signaling due to increase availability of the NOS substrate L-arginine. Another possibility is through an increase in polyamine metabolism since excessive arginase activity can increase the formation of polyamines via activation of the ornithine/polyamine pathway. It has been shown that excessive polyamine formation is involved in exitotoxic neuronal death in retina [32]. Further, polyamine metabolites have been shown to be involved in the pathogenesis of ischemic brain damage [36], [37], [38]. However, in the current study we have not investigated whether the regulation of NOS signaling or polyamine pathway is responsible for the neuroprotection in A2 deficient mice during OIR. Instead, our focus was on the apoptotic signaling involved in this process. Apoptosis can occur either through alterations in mitochondrial membrane permeability, release of cytochrome C and subsequent activation of caspases (intrinsic pathway) or via death receptor pathway (extrinsic pathway) involving FAS ligand or TNF [39]. Our data show that the major mechanism of apoptosis during OIR is through alterations in mitochondrial membrane permeability mediated by p53. Considering the predominant localization of A2 in mitochondria, our data showing reduced levels of Bim, cytochrome C and cleaved caspase 9 in A2−/− compared to WT during OIR suggest that neuronal apoptosis during ROP involves the intrinsic pathway. Further, targeting arginase 2 activity may provide a new therapeutic strategy for the prevention of neuro-glial injury in ROP patients.

## Materials and Methods

### Ethics statement

This study was carried out in strict accordance with the recommendations in the Guide for the Care and Use of Laboratory Animals of the National Institutes of Health. The protocol was approved by the Institutional Animal Care and Use Committee of the Georgia Health Sciences University. All surgery was performed under avertin (2, 2, 2 tribromoethanol, Sigma) anesthesia, and all efforts were made to minimize suffering.

### Antibodies

Antibodies were purchased from Santacruz (Arginase 2, Cellular Retinaldehyde-Binding Protein (CRALBP)), Sigma (Calbindin, Glial Fibillary Acidic Protein/GFAP, Tubulin), Abcam (Protein Kinase Cα/PKCα), Millipore (Recoverin) and Cell Signaling (P53, Cleaved Caspase 9, Cytochrome C, BCLxl and Bim).

### Animal model of OIR

WT controls (C57BL6) and mice deficient in arginase 2 (A2−/−) on C57BL6 background maintained in our animal facility were used for this study. A2*^−/−^* mice developed by Shi et al [40] were provided by Dr. Cederbaum (University of California, Los Angeles, California) with the permission of Dr. O'Brien. Homozygous arginase 2-deficient animals are fertile and do not have a distinguishable phenotype. The mouse model for OIR was generated according to the method of Smith et al [41] with minor modifications. Briefly, on P7 newborn mice are placed with their dam in 70% oxygen (hyperoxia) for 5 days and then returned to room air on P12. The hyperoxia treatment causes vaso-obliteration, but retinal development persists which results in relative retinal hypoxia when the mice are returned to room air. During the relative hypoxic period, the animals develop extensive retinal neovascularization and undergo glial cell activation and neuronal apoptosis. Mice were sacrificed at various times after the hyperoxia treatment and their retinae were collected and analyzed as described below.

### Morphometric analysis

Retinal morphology was studied on retinal cryostat sections from WT room air (WT RA), WT OIR and A2−/− OIR mice on P17. Sagital sections (10 um) containing the optic nerve were collected 20 um apart from one another, and stained with hematoxylin and eosin (H&E, Fischer Scientific). Digitalized images were taken 1000 µm from the optic nerve and four sections per animal were used. Thickness of the retina and inner nuclear layer (INL) was measured using NIH ImageJ.

### TUNEL assay

Apoptotic cells were studied using TUNEL (Terminal deoxynucleotidyl transferase dUTP nick end labeling) assay on cryosections (Fluorescein Insitu Cell death detection kit, Millipore) as per the manufacture's protocol. Quantification of TUNEL positive cells on retinal cryosection images was performed manually from optic disc to periphery. A minimum of four sections (20 um apart to one another) per animal were used.

### Immunohistochemistry

Eyes were enucleated, fixed in 4% paraformaldehyde (overnight, 4°C), washed in PBS and retinas were isolated and cryoprotected in 30% sucrose. Cryostat sections (10 um) were permeabilized in 1% Triton (20 min) and blocked in 10% normal goat serum containing 10% BSA (1 h). Sections were then incubated overnight in respective primary antibodies (4°C). On day two, they were incubated (1 h) in Flourescein or Texas red conjugated secondary antibodies (Molecular Probes), washed in PBS and mounted with Vectashield (Vector Laboratories).

### Retinal flatmount staining

Immunostaining on retinal flatmounts was performed as described previously [42] with minor modifications. Eyes were enucleated, fixed in 4% paraformaldehyde (overnight, 4°C), retinas were isolated washed in PBS and cut towards the optic disc to get a flower shape. The tissue was then permeabilized in 10% Triton X-100 for 20 min, blocked in 10% NGS/1% BSA/0.1% Triton X-100 and incubated in primary antibody overnight (4°C). The retinas were incubated in respective secondary antibodies for 4 h at 4°C, washed in PBS and mounted using Vectashield.

### Arginase activity

Arginase activity was measured using a colorimetric determination of urea production from L-arginine as described previously [43]. In brief, retinas were placed in Tris/EDTA buffer containing protease and phosphatase inhibitors and exposed to 3 freeze-thaw cycles and centrifuged at 14,000xg for 15 min at 4°C. Lysate (25 µl) was heated with MnCl_2_ (10^−3^ M) for 10 minutes at 56°C to activate arginase. The mixture was then incubated with 50 µL L-arginine (0.5 M, pH 9.7, 1 h, 37°C) to hydrolyze the L-arginine. The hydrolysis reaction was stopped with acid and the mixture was then heated at 100°C with 25 µL α-Isonitrosopropiophenone (9% α-ISPF in EtOH) for 45 minutes for the colorimetric reaction. The samples were kept in dark at room temperature for 10 minutes and absorbance was measured at 540 nm.

### Western blotting

Retinal homogenates were prepared using RIPA buffer (Millipore) containing protease and phosphatase inhibitors (complete mini and phosSTOP, respectively (Roche Applied Sciences). Proteins were separated on SDS-PAGE and transferred onto nitrocellulose membrane (Millipore), blocked in 5% milk or 3% BSA in TBST (Tris buffered saline with 0.5% Tween-20). The membrane was incubated overnight (4°C) in primary antibodies diluted in the blocking solution, washed and incubated in respective secondary antibodies (HRP conjugated, GE Healthcare) for 1 h at room temperature and developed using Enhanced Chemiluminescence (ECL,GE Health Care) or Westdura Detection (Pierce).

### Bipolar cell quantification

Cryostat sections from WT RA, WT OIR and A2−/−OIR at P17 were immunostained using protein kinase C alpha (PKCα, marker for rod bipolar cells) antibody as described above. Images from six sections (20 um apart) were taken 1000 µm from optic nerve and the number of bipolar cell bodies was quantified manually across a length of 750 µm.

### Electroretinogram

Mice were dark-adapted for 12 hours, anesthetized with Ketamine/Xylazine (80/16 mg/kg i.p.), and eyes were anesthetized and dilated (with Proparacaine, Tropicamide, and Phenylephrine). Signals were recorded via DTL electrodes on each eye, referenced to needle electrodes in the cheeks. Visual stimuli were generated by 5 ms duration flashes of a white LED. Responses were averaged across the two eyes for each mouse.

### Microscopy

Imaging of retinal cryostat sections was performed using a Zeiss Axioplan Imager Microscope and images taken using a Zeiss Axiocam digital camera, and Zeiss Axiovision 4.8.2 software. Retinal flatmounts were images using confocal microscope (Zeiss LSM 510 META).

### Statistical analysis

One-way ANOVA with posthoc test using GraphPad Prism software was performed in all analyses unless otherwise stated.
